# Ginsenoside Rg1 Promotes the Survival, Proliferation, and Differentiation of Senescent Neural Stem Cells Induced by D-galactose

**DOI:** 10.62641/aep.v53i1.1812

**Published:** 2025-01-05

**Authors:** Peiyu Sun, Shunhe Wang, Ling Hu, Yinhu Huang, Yaping Wang

**Affiliations:** ^1^Lab of Stem Cells and Tissue Engineering, Chongqing Medical University, 400016 Chongqing, China; ^2^Department of Pathology, Chongqing Medical University, 400016 Chongqing, China; ^3^Department of Histology and Embryology, Chongqing Medical University, 400016 Chongqing, China

**Keywords:** ginsenoside Rg1, D-galactose, neural stem cells, aging

## Abstract

**Background:**

Neural stem cells (NSCs) disrupt with aging, contributing to neurodegeneration. Ginsenoside Rg1 (Rg1), a compound found in Ginseng, is known for its anti-aging effects; however, its role in the progression of aging NSCs remains unclear. Therefore, this investigation explored the impact of Rg1 on the growth and maturation of aging NSC and elucidated its underlying molecular mechanisms.

**Methods:**

Initially, mouse models of brain aging were successfully established using D-galactose (D-gal) injection. Mice received Rg1 treatment along with D-gal administration. Brain tissues and NSCs were isolated and analyzed for pathological changes, gene expression, and cellular function. *In vitro*, experiments used aging NSCs treated with Rg1 to assess cell viability, proliferation, differentiation, and senescence markers.

**Results:**

D-gal triggered aging-related pathological alterations in mouse brains, elevated acetylcholinesterase levels, upregulated senescence genes, and inhibited NSC proliferation (*p* < 0.05). However, Rg1 treatment mitigated D-gal-induced effects, delayed brain aging, and improved NSC function. *In vitro*, Rg1 significantly increased cell viability, promoted NSC proliferation and differentiation, reduced senescent neurons, and downregulated *p53* and *p21* genes (*p* < 0.05).

**Conclusions:**

Rg1 demonstrates anti-aging properties in D-gal-induced mouse brain aging, promoting the proliferation and differentiation of NSCs, and downregulating the *p53*-*p21* signaling pathway.

## Introduction

Neurodegenerative diseases (NDDs) are a growing concern, particularly among the aging population, and pose a significant global health [1]. With the global population trending towards older demographics, there is a need to advance research on the underlying mechanisms and preventative strategies for NDDs [2]. Neural stem cells (NSCs), situated in the subventricular zone and subgranular zone of the adult nervous system, possess remarkable capabilities of self-renewal and multi-directional differentiation [3]. However, with increasing age, NSCs undergo gradual degeneration and senescence, contributing significantly to the onset and progression of NDDs [4, 5]. To address this problem, researchers have explored NSC transplantation as a potential therapeutic approach [6]. While animal models of brain aging have demonstrated promising results, translating these findings into effective human trials has been challenging [7]. Therefore, an alternative strategy to prevent and manage NDDs involves retarding the aging of autologous NSCs.

In this context, Ginsenoside Rg1 (Rg1), a key anti-aging compound found in the traditional Chinese herb Ginseng [8], has been focused on its potential to modulate NSC aging and brain senescence. Our previous studies have demonstrated that Rg1 can mitigate intracellular oxidative stress and alleviate free radicals production, subsequently reducing NSCs senescence resulting from deoxynucleic acid (DNA) damage and telomere shortening, thereby delaying nervous system aging [9, 10, 11, 12]. However, the underlying mechanisms remain unclear. Considering that impairments in NSC proliferation and differentiation are the primary causes of neurodevelopmental disorders [13], we hypothesize that the pharmacological mechanisms or pathways of Rg1 may involve regulating NSC proliferation and differentiation states. To gain further insights, we investigate the effects of Rg1 on the growth and differentiation of D-galactose (D-gal)-induced senescent NSCs. Our study particularly focuses on markers such as β-tubulin III (Tuj1), a neuronal lineage marker [14], glial fibrillary acidic protein (GFAP), an astrocytic marker [15], and 2^′^,3^′^-cyclic nucleotide 3^′^-phosphodiesterase (CNPase), a myelin-associated oligodendroglial marker [16], to assess the differentiation potential of NSCs. Additionally, we examine the potential involvement of *p21* and *p53*, two critical regulators of the cell cycle and apoptosis, in mediating Rg1’s regulatory effects on NSC aging [17].

## Materials and Methods

### Animal Treatment

Specific-pathogen-free (SPF) male C57BL/6 J mice (n = 40), aged 6–8 weeks and weighed 17 ± 1 g, were acquired from the Experimental Animal Center of Chongqing Medical University, China. They were housed in a controlled environment with unrestricted access to food and water.

Mice were randomly divided into four groups, and each group was treated as follows: the control group received intraperitoneal saline for 42 days; the Rg1 treatment group received intraperitoneal saline for 42 days, followed by intraperitoneal ginsenoside Rg1 (A0237; Chengdu Must Bio-Technology, Chengdu, China) at 40 mg/kg from day 16 for 26 days; the Rg1 plus D-gal treatment group received intraperitoneal D-gal (D8310; Solarbio Science & Technology, Beijing, China) at 200 mg/kg for 42 days, and intraperitoneal Rg1 at 40 mg/kg from day 16 for 26 days; the D-gal treatment group received intraperitoneal D-galactose at 200 mg/kg for 42 days.

Concerning the safety of Rg1 and D-gal, our previous studies have examined their effects on various organ systems [11, 18, 19], indicating no cytotoxicity or organ damage at these dosages. After treatment, all subject mice were euthanized following anesthesia, and brain tissues from each group were collected for subsequent analyses.

### Histopathologic Examination of Brain Tissue

Mice were euthanized using CO_2_ and deep anesthesia with pentobarbital (50 mg/kg), followed by perfusion with 4% buffered paraformaldehyde solution [20]. The brain tissues were then isolated, and the hippocampal region was selected for preparing frozen and paraffin sections. Histological changes were assessed utilizing hematoxylin and eosin (HE) and Nissl staining. Neuronal cell density was determined from three randomly selected sections spanning the central portion of the dentate gyrus (DG) region. Neuronal cell density was calculated as follows: Neuronal cell density = Area of neuronal cell nucleus/Area of statistical field. The number of Nissl bodies in the DG region was evaluated using the average optical density (AOD), which is calculated as: AOD = Integrated optical density/Area of statistical field.

### Immunohistochemistry of Brain Tissue

The paraffin-embedded sections were dewaxed and rehydrated. Antigen retrieval was conducted by boiling these sections in a citrate buffer. To neutralize endogenous peroxidase, they were exposed to 3% H_2_O_2_ for 20 minutes. They were then blocked utilizing 10% bovine serum albumin (BSA) for 1 hour at ambient temperature followed by overnight incubation at 4 °C with primary antibodies against Nestin (1:100; AT809; Beyotime Biotechnology, Shanghai, China) and Ki-67 (1:500; GB111141; Servicebio, Wuhan, China). After rinsing, the tissue sections were incubated with HRP-linked secondary antibodies (1:3000; 7074, 7076; Cell Signaling Technology, Danvers, MA, USA) for 1 hour at ambient temperature. In the next step, these tissue sections were developed with diaminobenzidine (DAB) for 3 minutes, washed, and mounted. The number of Nestin^+^ cells or Ki-67^+^ cells was determined by counting at least three arbitrarily selected slices from the central portion of the DG region.

### Detection of Acetylcholinesterase Activity in Supernatants of the Hippocampus

Hippocampal tissues were harvested and lysed in an ice-cold environment for 30 minutes. The samples were centrifuged at 8000 g and 4 °C for 10 minutes, and the supernatant fluid was collected. Acetylcholinesterase (AChE) activity was assessed using a microtiter plate reader following the manufacturer’s protocol (BC2025; Solarbio Science & Technology, Beijing, China). AChE catalyzes the hydrolysis of acetylcholine, yielding choline, which reacts with dithio-p-nitrobenzoic acid to generate 5-mercapto-nitrobenzoic acid (TNB). One unit of AChE activity is the production of 1 nmol TNB per mg of tissue protein per minute, expressed as U/mg prot/min. AChE activity was determined utilizing the following formula: AChE activity (U/mg prot) = (detection group OD – control group OD) × 2255/0.6/Cpr (mg protein/mL).

### NSCs Culture and Treatment

Neonatal SPF C57/BL/6 J mice (n = 10), aged 1 day and weighed 1–2 g, were submerged in 75% alcohol for 3 minutes. The animals were euthanized by decapitation, and their craniums were carefully dissected and placed in sterilized containers. All cerebral tissues were extracted into a vessel containing tissue harvesting solution, finely minced, and disaggregated into individual cells utilizing Accutase^TM^ (07920; STEMCELL Technology, Vancouver, BC, Canada). The cell suspension was then centrifuged to remove the supernatant and cultured using a serum-free neural progenitor cell proliferation medium (MUXNF-90011; Cyagen Biosciences, Guangzhuou, China). These cultures were regularly examined for mycoplasma contamination (G1902; Servicebio, Wuhan, China) and confirmed negative. Moreover, to identify P3 generation NSCs, immunofluorescence staining for the neural stem cell-specific markers Nestin (1:25; AN205; Beyotime Biotechnology, Shanghai, China) and SOX2 (1:200; BM4147; Boster Biological Technology, Wuhan, China) was performed.

P3 generation NSCs were randomly categorized into four groups: the control, Rg1 treatment, Rg1 plus D-gal treatment, and D-gal treatment groups. The D-gal treatment group was incubated with D-gal (10 mg/mL) for 48 hours. In the Rg1 plus D-gal treatment group, Rg1 (20 µg/mL) was administrated in combination with the D-gal treatment for 48 hours. The Rg1 treatment group was cultured with Rg1 (20 µg/mL) for 48 hours. The control group underwent standard cultivation for 48 hours. Aging-related indicators were evaluated after the drug interventions, and differentiated culture of NSCs was simultaneously conducted in parallel.

### NSCs Differentiation Assay

After treatment of each NSCs group, the serum-free medium was substituted with the differentiation medium (DMEM/F12 medium containing 2% B27 and 1% fetal bovine serum). Cells were seeded in 24-well or 6-well culture plates coated with poly-L-lysine at a concentration of 5 × 10^4^ cells/mL and incubated for 7 days. After this, the cells in the 24-well cell culture plates were fixed with 4% paraformaldehyde solution for 20 minutes for immunofluorescence analysis. Moreover, cells in the 6-well plates were lysed with RNAiso Plus (9108; TaKaRa, Beijing, China), and the samples were collected and stored at –80 °C for subsequent real-time quantitative polymerase chain reaction (RT-qPCR) analysis.

### NSCs Proliferative and Cell Viability Assay

The treated NSCs were seeded in 96-well plates with 100 µL culture medium at 1 × 10^4^ cells per well. Cell proliferation was then assessed at 0, 12, 24, 36, and 48 hours using the Cell Counting Kit-8 (CCK-8), with three replicate wells in each experimental condition. After this, 10 µL CCK-8 reagent was added to each well and was incubated for 2 hours. Finally, the optical density (OD) at 450 nm was determined using a microtiter plate reader.

Furthermore, cell viability was assessed utilizing the trypan blue exclusion method. After treatment, NSCs from each group were digested into single cells and resuspended in a culture medium. Subsequently, equivalent volumes of the cell suspension and trypan blue solution were mixed and employed for cell counting with a hemocytometer. Cells that stained blue were categorized as nonviable, while unstained cells were considered viable. Finally, the proportion of viable cells was computed by dividing the count of viable cells by the total number of cells.

### Senescence Associated β-galactose (SA-β-gal) Staining

The senescent condition of the cells was assessed using the SA-β-gal staining Kit (C0602; Beyotime Biotechnology, Shanghai, China). Briefly, cryosections and neurospheres were fixed for 30 minutes at ambient temperature, rinsed three times with phosphate-buffered saline (PBS) and incubated with the fresh β-galactosidase staining solution at 37 °C for 12 hours. Post-staining, the specimens were rinsed three times with PBS and examined under a light microscope. The total number of SA-β-gal-positive neurospheres was determined by counting 100 randomly selected neurospheres. The AOD was quantified to indicate the proportion of SA-β-gal positive NSCs in the sections.

### Immunofluorescence Analysis

For immunofluorescence analysis, appropriate frozen sections were fixed in 4% paraformaldehyde (PFA, P0099-500mL, Beyotime Biotechnology, Shanghai, China) solution for 30 minutes, washed with PBS, and permeabilized with 0.2% Triton X-100 (P0096-100mL, Beyotime Biotechnology, Shanghai, China) for 10 minutes. The tissue sections were then blocked with 10% BSA for 1 hour at ambient temperature. After this, the tissue sections were incubated with antibodies against Tuj1 (1:250; AT809; Beyotime Biotechnology, Shanghai, China), GFAP (1:100; AG259; Beyotime Biotechnology, Shanghai, China), and CNPase (1:100; A1018; ABclonal, Wuhan, China) for 12 hours at 4 °C. After washing with PBS, the sections were exposed to fluorescein isothiocyanate (FITC) or cyanine 3 (Cy3) labelled secondary antibodies (1:200; GB21303, GB22401, GB22403; Servicebio, Wuhan, China) at 37 °C for 1 hour, followed by nuclear staining with 4^′^,6-diamidino-2-phenylindole (DAPI, C1005; Beyotime Biotechnology, Shanghai, China) for 5 minutes. Following PBS washes, glycerol 20 µL was applied to each slide, and a coverslip was placed on top. Each slide was analyzed using a fluorescence microscope to calculate the relative fluorescence intensity, which was assessed as follows: Relative fluorescence intensity = fluorescence intensity of FITC or Cy3/fluorescence intensity of DAPI.

Furthermore, cells in the 24-well plates underwent a similar immunofluorescence staining procedure. The proportion of positive cells in the 24-well plates was ascertained by assessing the ratio of FITC^+^ or Cy3^+^ cells to DAPI^+^ cells within the same field of view.

### Western Blot Analysis

Total protein was extracted from the hippocampal tissue of each group using radio-immunoprecipitation assay (RIPA) Lysis Buffer and subsequently quantified employing the BCA protein assay kit. The proteins were resolved by sodium dodecyl sulfate-polyacrylamide gel electrophoresis (SDS-PAGE) and then transferred to polyvinylidene difluoride (PVDF) membranes. The membranes were then blocked with 5% non-fat milk in tris-buffered saline tween (TBST) for 2 hours at ambient temperature. After this, the membranes were incubated with primary antibodies against glyceraldehyde 3-phosphate dehydrogenase (GAPDH), Tuj1, GFAP, CNPase, p53 (BF8013; Affinity Biosciences, Changzhou, China), and p21 (AF6290; Affinity Biosciences, Changzhou, China), all diluted at 1:1000, for 12 hours at 4 °C. After rinsing with TBST, the membranes were incubated with secondary antibodies (1:2000; 7074, 7076; Cell Signaling Technology, Danvers, MA, USA) for 1 hour at 37 °C. Protein bands were visualized utilizing the ChemiDoc System (BIO-RAD, Hercules, CA, USA). GAPDH was used as the internal control, and relative protein intensities were assessed using ImageJ (1.46r, Bethesda, MD, USA) software.

### RNA Extraction and RT-qPCR

Cells in the 6-well plates were collected, and total ribonucleic acid (RNA) was extracted using RNAiso Plus following the manufacturer’s protocol. RNA concentration and purity were examined using a spectrophotometer. In the next step, RNA was converted into cDNA employing the PrimerScript RT reagent Kit (RR047A; TaKaRa, Beijing, China). Amplification of the cDNA was performed with the CFX Connect Real-Time PCR Detection System (BIO-RAD, Hercules, CA, USA). Thermocycler conditions were set as follows: initial denaturation at 95 °C for 3 minutes, followed by 40 cycles of 95 °C denaturation (5 seconds) and 60 °C annealing and extension (30 seconds). mRNA expression levels were normalized against *GAPDH* mRNA and evaluated using the 2^-Δ⁢Δ⁢Ct^ method. A list of primers used in RT-qPCR is shown in Table 1.

**Table 1.** **A list of primers used in RT-qPCR**.

Genes	Forward (5^′^-3^′^)	Reverse (5^′^-3^′^)
*Tuj1*	CAGCGATGAGCACGGCATAGAC	CCAGGTTCCAAGTCCACCAGAATG
*GFAP*	AGATTCGCACTCAATACGAGG	CTGTGAGGTCTGCAAACTTAGA
*CNPase*	CTTCAAGAAAGAGCTTCGACAC	CAGAATTTGGTTGTACAGTGCA
*p21*	CCTTGTCGCTGTCTTGCACTCTG	GCTGGTCTGCCTCCGTTTTCG
*p53*	ACCGCCGACCTATCCTTACCATC	GGCACAAACACGAACCTCAAAGC
*GAPDH*	TGACGTGCCGCCTGGAGAAA	AGTGTAGCCCAAGATGCCCTTCAG

*Tuj1*, β-tubulin III; *GFAP*, glial fibrillary acidic protein; *CNPase*, 2^′^,3^′^-cyclic nucleotide 3^′^-phosphodiesterase; *GAPDH*, glyceraldehyde 3-phosphate dehydrogenase; RT-qPCR, real-time quantitative polymerase chain reaction.

### Statistical Analysis

Statistical analysis was performed using GraphPad Prism 8.3.0 (GraphPad Software, San Diego, CA, USA). Empirical data were expressed as the mean ± standard deviation (SD). Comparison between the two groups was performed using Student’s *t*-test, and multiple group comparisons were assessed using analysis of variance (ANOVA). A *p*-value < 0.05 indicated statistical significance.

## Results

### Rg1 Reduces D-gal-induced Hippocampal Neuron Damage in Aging Mice

**Fig. 1.**
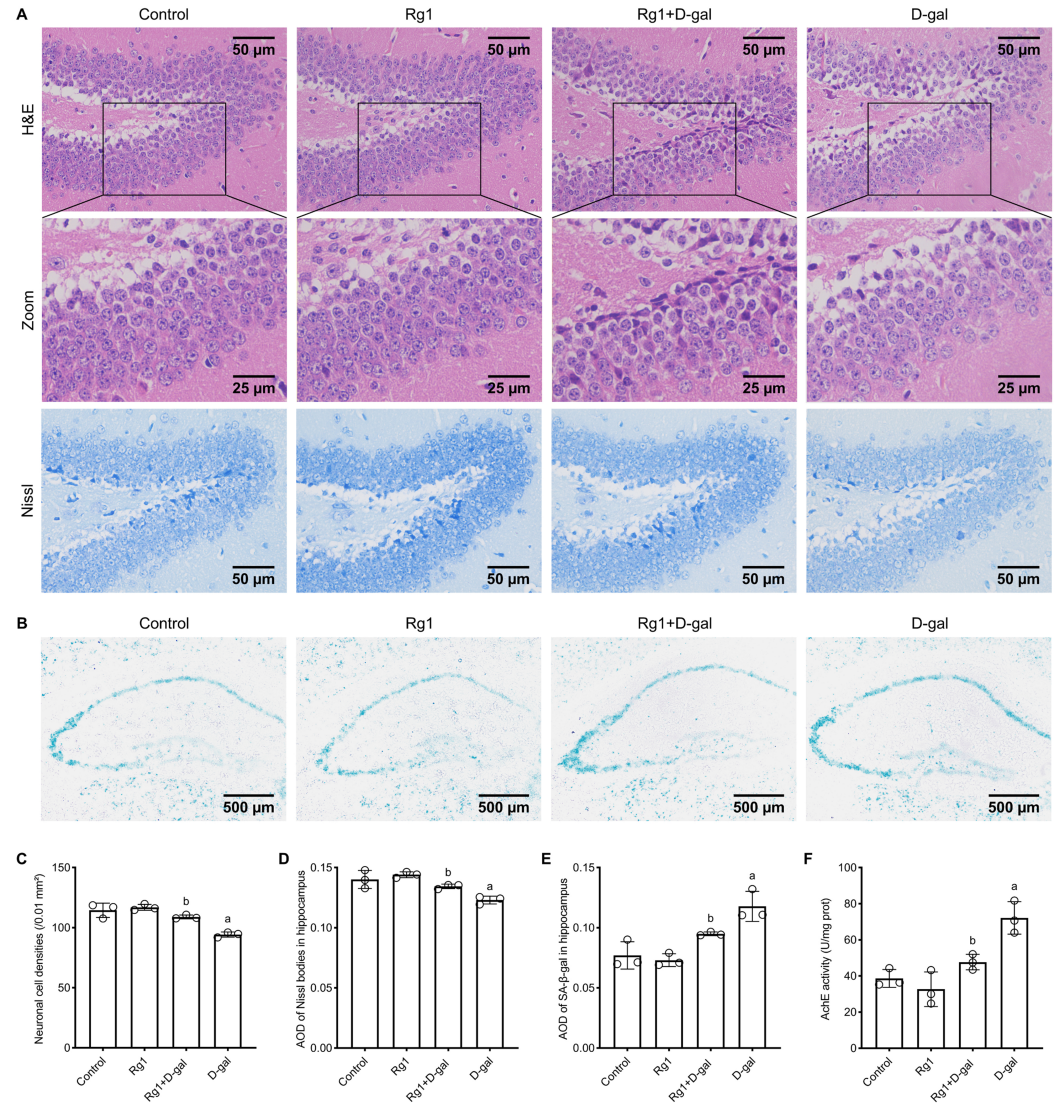
**Ginsenoside Rg1 reduces aging in brain senescence mice**. (A) Hematoxylin and eosin (H&E) staining and Nissl staining were employed to examine histopathological changes and assess hippocampal neuron damage in brain senescence mice. (B) Senescence-associated β-galactose (SA-β-gal) staining of the frozen sections elevated the aging of the hippocampal tissues. The cytoplasm was stained blue in aging cells. (C) Statistical plot of neuronal cell densities in HE-stained sections. (D) Statistical plot of average optical density (AOD) of Nissl bodies in the hippocampus in Nissl-stained sections. (E) Statistical plot of AOD of SA-β-gal in the hippocampus in SA-β-gal-stained sections. (F) Evaluation of acetylcholinesterase activity in supernatants of hippocampal tissues. The data are presented as mean ± SD (n = 3 per group). ^a^*p* < 0.05 vs. Control; ^b^*p* < 0.05 vs. D-gal. SD, standard deviation.

Hematoxylin and eosin (H&E) staining revealed that, compared to the control group, the hippocampal neurons in the D-gal treatment group were disorganized, sparsely distributed, and showed morphological abnormalities, including an increased number of degenerated and necrotic cells. Furthermore, neuronal density was substantially diminished. However, in the Rg1 plus D-gal treatment group, the hippocampal neuronal density did not exhibit a substantial reduction relative to the D-gal group, and no significant morphological damage was observed. Moreover, Nissl staining demonstrated that, relative to the control group, the staining intensity of Nissl bodies in the hippocampal neurons of the D-gal group was significantly alleviated. Notably, compared to the D-gal group, the Rg1 plus D-gal treatment group displayed a significant enhancement in Nissl body staining intensity in the hippocampal neurons (Fig. 1A,C,D). These findings indicate that Rg1 can alleviate D-gal-induced hippocampal neuronal damage in mice.

### Rg1 Decreases AChE Activity in the Hippocampus of Brain-aging Mice

During the aging, AChE plays a crucial function in modulating synaptic transmission by the hydrolysis of the neurotransmitter acetylcholine [21]. We observed that AChE activity in the hippocampal tissue homogenate of the D-gal treatment group was substantially elevated compared to the control group. However, compared to the D-gal treatment group, AChE activity was significantly reduced in the Rg1 plus D-gal treatment group (Fig. 1F). These outcomes indicate that the neuroprotective effect of Rg1 against D-gal-induced neuronal damage is associated with the inhibition of AChE activity in the hippocampus.

### Culture and Identification of NSCs

**Fig. 2.**
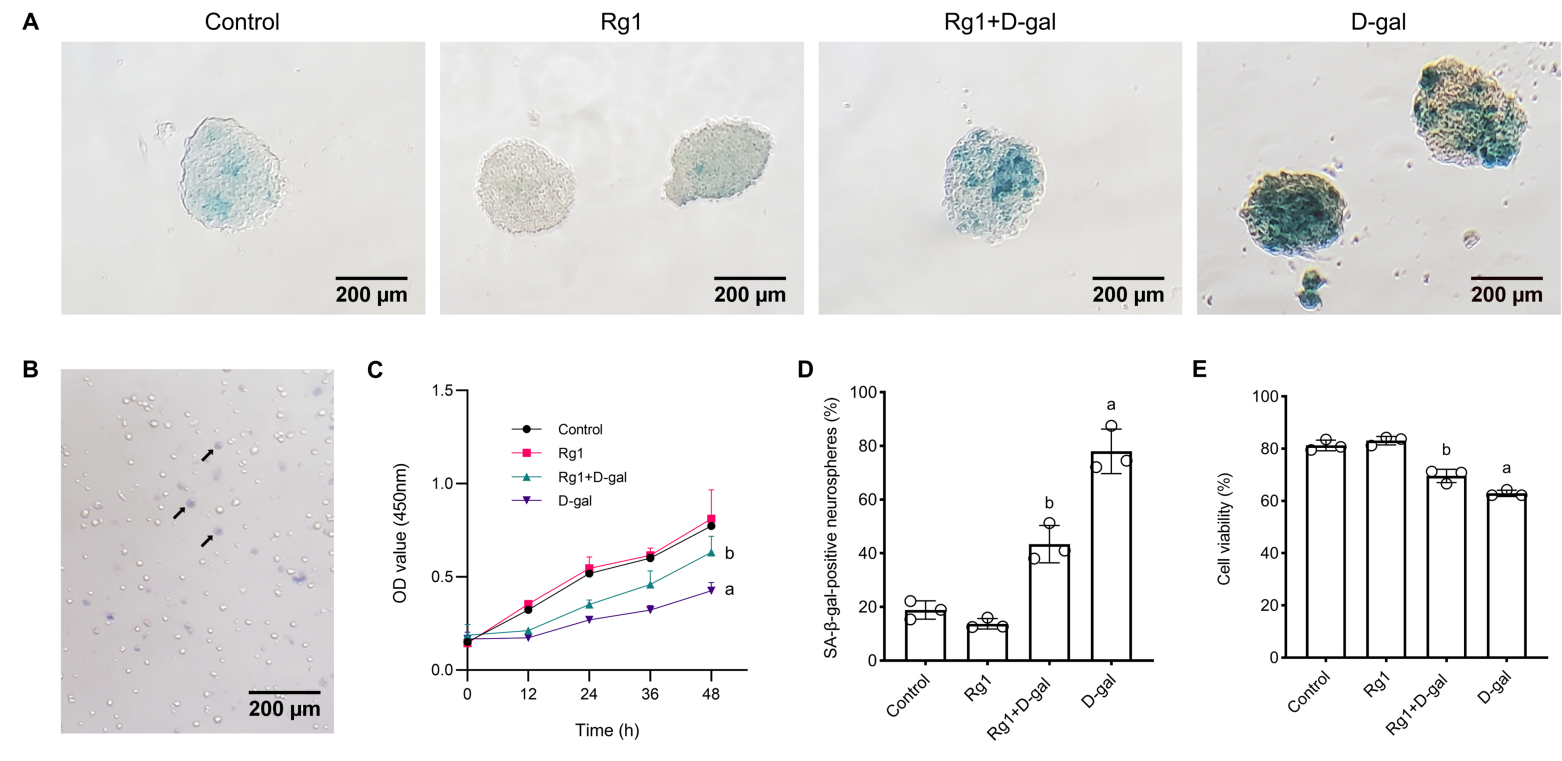
**Ginsenoside Rg1 slowed the aging of neural stem cells *in vitro***. (A) The neural stem cells (NSCs) were cultured after treatment until forming neurospheres on day-7. The senescence associated β-galactose (SA-β-gal) staining was used to elevate the aging of NSCs. The cytoplasm was stained blue in aging NSCs. (B) Cell viability was examined using trypan blue exclusion assay. Arrows indicate trypan blue-positive NSCs. (C) The Cell Counting Kit-8 (CCK-8) method was used to assess the proliferation and viability of NSCs. (D) Statistical plot of the percentage of SA-β-gal staining positive neurospheres. (E) Statistical plot of cell viability. The data are expressed as mean ± SD (n = 3 per group). ^a^*p* < 0.05 vs. Control; ^b^*p* < 0.05 vs. D-gal.

NSCs were successfully isolated from mouse brain tissue and cultured, forming three-dimensional structural neurospheres after 7 days (Fig. 2A). Immunofluorescence staining confirmed that the P3 generation neurospheres expressed both Nestin and SOX2. After 7 days of culture in the differentiation medium, the neurospheres began to adhere and differentiate, indicating that the passaged NSCs maintained their self-renewal and multipotent characteristics. Therefore, these purified NSCs were used for subsequent analysis.

### Rg1 Promotes Proliferation and Viability of Aging NSCs in Vitro

To examine the impact of Rg1 treatment on the proliferation and viability of aging NSCs induced by D-gal, NSCs were cultured in 96-well plates and evaluated using the CCK-8 assay. After 48 hours of incubation, the OD values for the D-gal treatment group were significantly diminished compared to the control group. However, the OD values of the Rg1 plus D-gal treatment group exhibited a substantial increase compared to the D-gal treatment group. These findings indicate that Rg1 can improve the proliferation viability of aging NSCs (Fig. 2C).

Dead cells are differentiated from live cells by their inability to exclude trypan blue due to membrane impermeability [22] (Fig. 2B). We observed that the viability of NSCs in the D-gal-treated group was substantially reduced compared to the control group. Conversely, the Rg1 plus D-gal treatment group showed an elevated NSCs viability compared to the D-gal treatment group (Fig. 2E).

### Rg1 Attenuates D-gal-induced NSCs Senescence in Vitro and in Vivo

SA-β-gal is commonly used to identify aging cells [23], staining the cytoplasm of aged cells blue. Microscopic examination indicated blue-stained cells in the hippocampus and NSCs (Fig. 1B, Fig. 2A). We observed a significant increase in the proportion of senescent NSCs in the D-gal treatment group compared to the control group, as shown by the quantification of SA-β-gal-positive NSCs in both the hippocampus and* in vitro* conditions. The Rg1 plus D-gal treatment group demonstrated a substantial reduction in the percentage of senescent NSCs, indicating that Rg1 provides protective effects against NSC senescence (Fig. 1E, Fig. 2D).

### Rg1 Promotes the Differentiation of Aging NSCs in Vitro

**Fig. 3.**
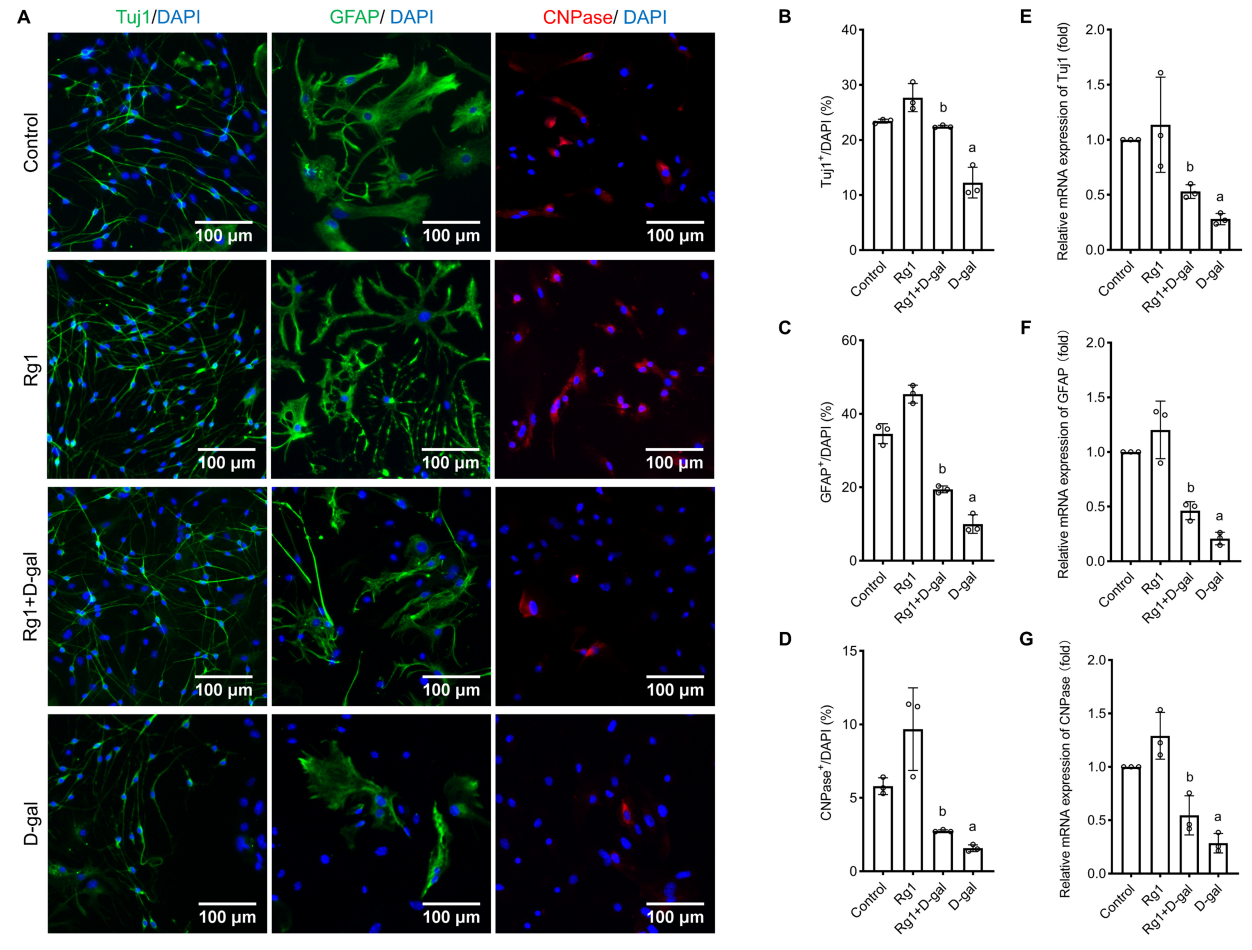
**Ginsenoside Rg1 promotes multi-directional differentiation of aging neural stem cells *in vitro***. (A) Immunofluorescence staining was used to detect the differentiation of neural stem cells (NSCs) into Tuj1^+^ neurons, GFAP^+^ astrocytes, and CNPase^+^ oligodendrocytes. (B–D) The relative proportion of NSCs differentiated into Tuj1^+^ neurons, GFAP^+^ astrocytes, and CNPase^+^ oligodendrocytes in each group. (E–G) The gene expression levels of *Tuj1*, *GFAP*, and *CNPase* after 7 days of differentiation NSCs *in vitro* in each cohort. The data are expressed as mean ± SD (n = 3 per group). ^a^*p* < 0.05 vs. Control; ^b^*p* < 0.05 vs. D-gal. DAPI, 4’6-diamidino-2-phenylindole.

Immunofluorescence staining was used to identify differentiated Tuj1^+^ neurons, GFAP^+^ astrocytes, and CNPase^+^ oligodendrocytes in the 24-well plates (Fig. 3A). After this, we calculated and statistically analyzed the percentages of Tuj1^+^/DAPI, GFAP^+^/DAPI, and CNPase^+^/DAPI in each group. Additionally, RT-qPCR was performed to determine *Tuj1*, *GFAP*, and *CNPase* mRNA expression in each group after 7 days of differentiation. The immunofluorescence staining and statistical analysis indicated that, compared to the control group, the D-gal treatment group exhibited significantly diminished percentages of neurons, astrocytes, and oligodendrocytes after 7 days of differentiation. Conversely, the Rg1 plus D-gal treatment group showed substantially elevated percentages of neurons, astrocytes, and oligodendrocytes compared to the D-gal treatment group (Fig. 3B–D). The results of RT-qPCR were consistent with the immunofluorescence findings (Fig. 3E–G). These findings demonstrate that Rg1 can promote the differentiation of aging NSCs into neurons, astrocytes, and oligodendrocytes.

**Fig. 4.**
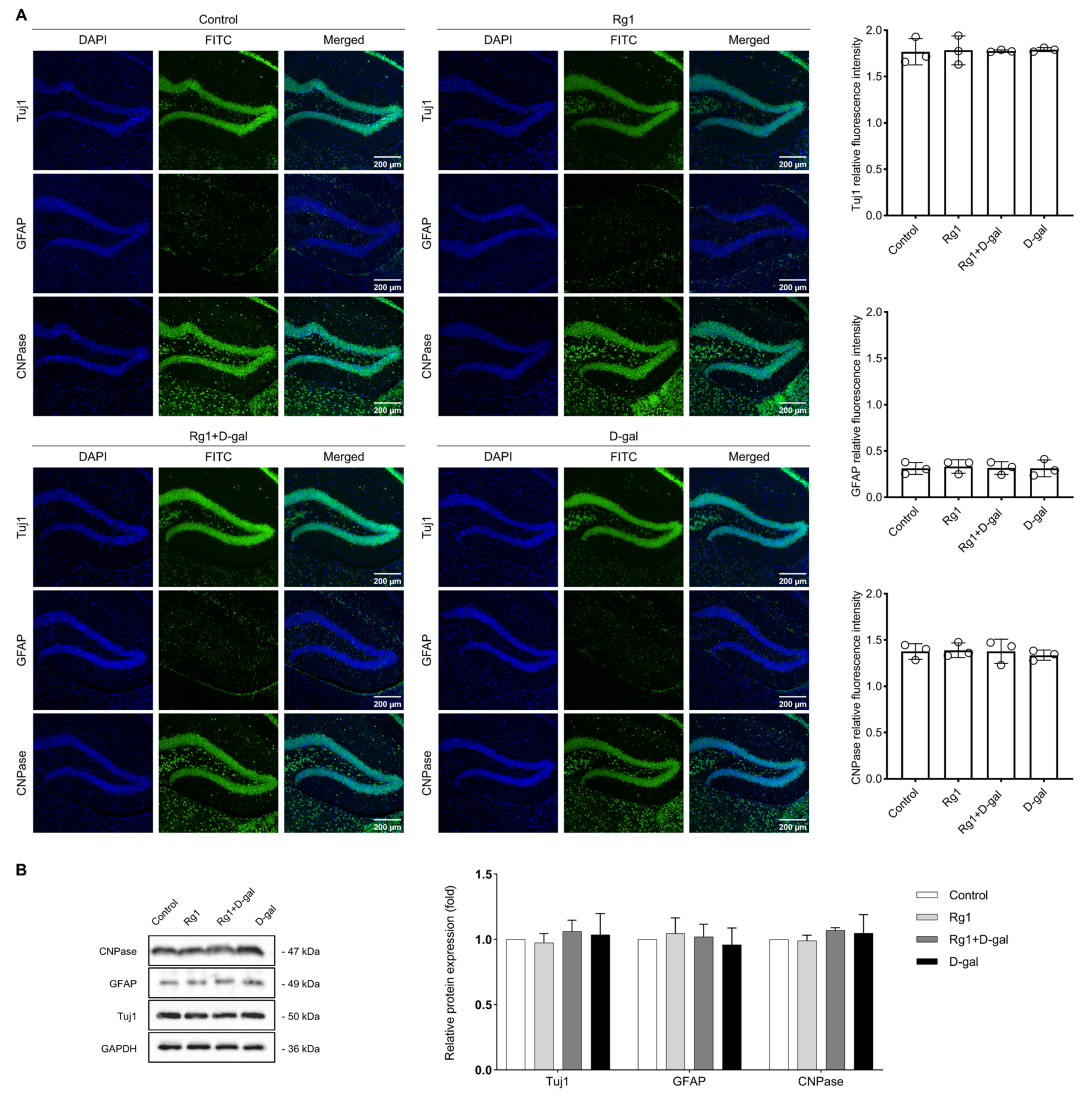
**Effects of ginsenoside Rg1 on the neural stem cells the differentiation in hippocampal dentate gyrus of brain senescence mice**. (A) Immunofluorescence staining was used to detect the relative fluorescence intensity of Tuj1^+^ neurons, GFAP^+^ astrocytes, and CNPase^+^ oligodendrocytes in each group of hippocampal sections. DAPI fluorescence was used to normalize FITC fluorescence quantified for each section. The results are presented on the right side. (B) Western blot analysis of Tuj1, GFAP, and CNPase expression in the hippocampal dentate gyrus. The data are expressed as mean ± SD (n = 3 per group). There was no significant difference among the four groups (*p* > 0.05). FITC, fluorescein isothiocyanate.

Immunofluorescence was also performed on frozen sections, where the relative fluorescence intensities of Tuj1, GFAP, and CNPase were measured to assess the NSCs differentiation in the DG region of the hippocampus. DAPI fluorescence was used to normalize FITC fluorescence for each quantified hippocampal section. However, this method showed no substantial variations among the groups (Fig. 4A). Furthermore, Western blot analysis validated the immunofluorescence findings and yielded similar results (Fig. 4B). Therefore, we continued to assess this study from diverse perspectives in the field of aging research.

### Rg1 Promotes Survival and Proliferation of NSCs in Brain-aging Mice to Increase Neurogenesis

**Fig. 5.**
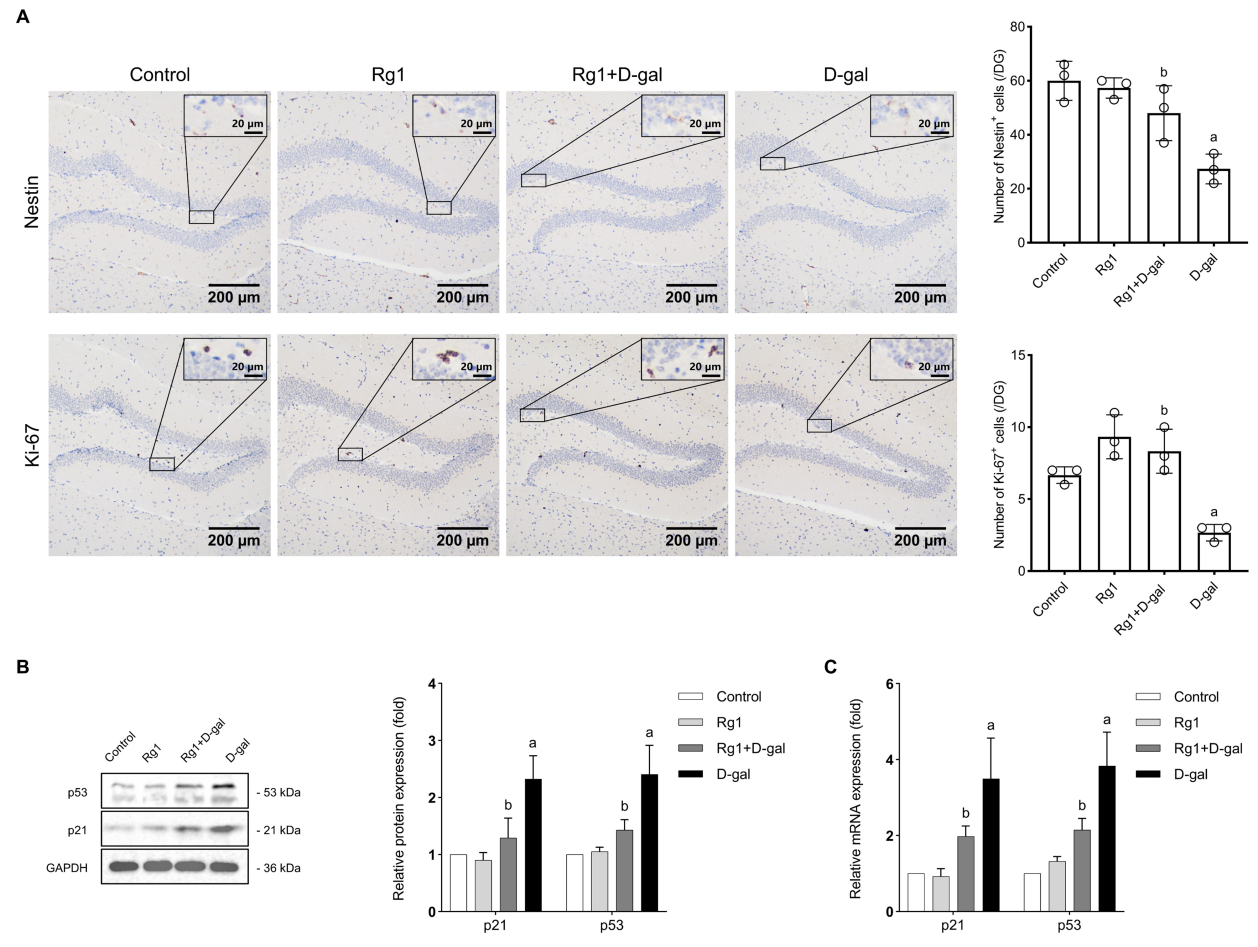
**Mechanism of ginsenoside Rg1 in delaying brain senescence of mice**. (A) Immunoreactivity of Nestin and Ki-67 in the hippocampal dentate gyrus (DG). The Nestin-immunoreactive somas and processes were designated as tan or brown. Most Ki-67-immunopositive cells were frequently clustered together with brown nuclei. The bar graphs on the right show the number of Nestin-immunopositive cells/DG and Ki-67-immunopositive cells/DG. (B) Western blot analysis of p21 and p53 expression levels in the hippocampal dentate gyrus. (C) Real-time quantitative PCR analysis of *p21* and *p53* expression levels in the NSCs. The data are presented as mean ± SD (n = 3 per group). ^a^*p* < 0.05 vs. Control; ^b^*p* < 0.05 vs. D-gal.

NSCs in the DG are vital to the hippocampus due to their ability to produce neurons [24]. In this study, we examined the number of NSCs in the hippocampus using immunohistochemical staining with the Nestin antibody [25]. A substantial reduction in the count of Nestin-positive NSCs was found in the D-gal treatment group. Conversely, the Rg1 plus D-gal treatment group exhibited a significantly higher number of NSCs, suggesting that Rg1 enhances the population of NSCs in the hippocampus of mice undergoing brain aging (Fig. 5A).

Ki-67 is a nuclear protein that manifests in all phases of the cell cycle except the quiescent stage, making it a reliable indicator of cell proliferation [26]. To assess cell proliferation in the hippocampus, we conducted immunohistochemical staining for Ki-67. The number of Ki-67^+^ cells was considerably lower in the D-gal treatment group than in the control cohort, suggesting that D-gal reduced cell proliferation in the hippocampus. Nonetheless, the Rg1 plus D-gal treatment group had substantially higher cell proliferation than the D-gal treatment group (Fig. 5A). These findings indicate that Rg1 attenuates D-gal-reduced cell proliferation in the hippocampus.

### Rg1 Down-regulates the Expression of Senescence-associated Genes in Vitro and in Vivo

The *p53*-*p21* pathway is a crucial signal transduction pathway in cell aging [17]. To elucidate this pathway, we used Western blotting to investigate the protein expressions of p53 and p21 in the hippocampus, a central component of this pathway. As shown in Fig. 5B, the expression levels of both p53 and p21 proteins were significantly higher in the D-gal-treated group compared to the control group. Conversely, the Rg1 plus D-gal treated group exhibited a significant reduction in these protein levels relative to the D-gal treated group. Additionally, we performed RT-qPCR to investigate the mRNA expression levels of *p53* and *p21 in vitro,* and the findings aligned with the Western blot results (Fig. 5C). These observations indicate that Rg1 can attenuate the expression of age-related genes in the hippocampus of brain-aging mice and NSCs.

## Discussion

In this study, we present novel evidence that Rg1 effectively mitigates neuronal injury induced by D-gal, suggesting its potential as a viable therapeutic agent for treating NDDs. Our findings indicate that Rg1 modulates the proliferation and function of hippocampal NSCs while negatively regulating the expression of aging-related genes, thereby delaying brain senescence. This study not only advances our understanding of Ginseng’s “Qi-tonifying and marrow-generating” properties but also offers potential therapeutic avenues for combating the growing challenge of NDDs. These findings highlight the significance of Rg1 in the treatment and prevention of NDD, underscoring the need for further investigation to fully explore its therapeutic potential.

D-galactose, a well-established oxidative aging agent, elicits a range of cellular damages, including oxidative stress, inflammatory responses, and apoptosis, resulting in an aging phenotype similar to natural aging [27]. Consequently, it is frequently employed in constructing aging models in animals and *in vitro* cells [28, 29]. In this study, we successfully established a mouse model of brain aging using the optimal D-gal dosage and duration previously identified by our research group [18]. Our findings revealed that mice in the D-gal cohort exhibited disrupted neuronal arrangement, decreased neuronal density, lower Nissl body counts, and increased SA-β-Gal positive cells in the DG of the hippocampus, all of which are morphological hallmarks of cellular aging. AChE regulates the speed and intensity of neural transmission by hydrolyzing the neurotransmitter acetylcholine into acetate and choline, thereby ensuring coordination and balance among various systems [21]. In this study, we observed increased AChE activity in the brain tissue of D-gal-treated mice, consistent with the manifestation of brain aging. Intervening with Rg1 via intraperitoneal injection during the D-gal-induced aging process, we found that Rg1 significantly mitigated the morphological damage to hippocampal neurons, diminished the number of SA-β-gal positive cells, and decreased AChE activity in brain-aged mice. Furthermore, we isolated and cultured NSCs from the whole brains of C57BL/6J mice. SA-β-gal staining, trypan blue exclusion assay, and CCK-8 cell proliferation assays demonstrated that Rg1 promoted NSC proliferation, enhanced NSC viability, and delayed NSC aging. These findings indicate that Rg1 can delay brain aging in mice and counteract D-gal-induced NSC aging *in vitro*, suggesting its potential for preventing and treating NDDs.

Neurogenesis in the hippocampal DG involves crucial processes such as the differentiation of NSCs into neurons, synaptic remodeling, and neuronal migration, all of which are essential for the normal function of the DG and for advanced cognitive functions like memory and learning [24, 30]. This functionality is intimately linked to the self-renewal of NSCs and the development of mature, new neurons in the DG, mediating numerous aspects of brain function [24, 31, 32]. One of the primary causes of neurodevelopmental disorders is the decline in the proliferative and differentiative capabilities of NSCs, and aging acts as a negative regulator of NSC self-replication and multipotency [33, 34]. The progressive loss of NSC regenerative potential leads to brain tissue degeneration and dysfunction, ultimately contributing to various neurodegenerative diseases of the central nervous system, such as Parkinson’s disease and Alzheimer’s disease [35]. Consequently, we hypothesize that the anti-aging effects of Rg1 on the brain may be associated with its ability to promote NSC proliferation and differentiation in the hippocampus.

In our *in vitro* model, the number of NSCs differentiating into neurons, astrocytes, and oligodendrocytes was substantially elevated in the Rg1 plus D-gal group relative to the D-gal group, indicating that Rg1 enhances the multipotency of NSCs. However, we did not observe any differences in NSC differentiation in the hippocampal DG region across the groups, suggesting that Rg1 does not significantly impact NSC differentiation in the brains of aged mice. This finding contrasts with a previous study that reported Rg1’s ability to promote the differentiation of aging NSCs into neurons [10]. This discrepancy may be attributed to differences in the source and purity of the drug, as well as the distinct animal models employed [36].

To further elucidate the mechanisms underlying Rg1’s delay of brain aging, we investigated its effects on NSC quantity and self-renewal capacity in the mouse hippocampus. Our results revealed that Rg1 significantly elevated the number of Nestin^+^ and Ki-67^+^ cells in the hippocampus of aged mice, suggesting that Rg1 can counteract age-related NSC loss and stimulate NSC proliferation.

Cell senescence can be triggered by various cell signaling pathways, including the *p53*-based pathway [17]. The accumulation of the p53 protein activates distinct gene expression profiles, resulting in G1 and G2 cell cycle arrest and preventing cell proliferation [37]. DNA damage can upregulate p53 protein levels via phosphorylation [38], enhancing its transcriptional activity. P21 is a major effector of p53 activation [39]. In this study, the observed reduction in *p53*, *p21* genes, and protein levels with Rg1 treatment suggests that Rg1 may maintain NSC proliferation and delay hippocampal aging by inhibiting the downstream *p53*-*p21* signaling pathway, regulating cell cycle progression, and modulating apoptosis. The statistical results of Nestin^+^ and Ki-67^+^ cell counts in the hippocampal DG region of each group provided support for this hypothesis.

The restricted regenerative ability of the mature nervous system means that neuronal loss or permanent impairment can induce the progression of NDDs. While conventional therapeutic agents like MDA receptor antagonists, levodopa, and donepezil can effectively manage symptoms by replenishing neurotransmitters and neurotrophic factors [40, 41, 42], their prolonged usage is often constrained by various adverse effects [43, 44]. In this context, Rg1 emerges as a promising and safe therapeutic candidate, offering a promising avenue for NSCs-based therapies by promoting adult neurogenesis, fostering NSC proliferation, and replenishing depleted or deficient cells in neurodegenerative conditions.

## Conclusions

In summary, our findings show that Rg1 effectively mitigates the neuronal injury induced by D-gal, suggesting its potential as a viable therapeutic agent for treating NDDs. Furthermore, we propose that Rg1 modulates the proliferation and function of hippocampal NSCs while negatively regulating the expression of aging-related genes, thereby delaying brain senescence.

## Availability of Data and Materials

The data used to support the findings of this study are available from the corresponding author upon reasonable request.
